# The Use of Intravenous Antibiotics at the Onset of Neutropenia in Patients Receiving Outpatient-Based Hematopoietic Stem Cell Transplants

**DOI:** 10.1371/journal.pone.0046220

**Published:** 2012-09-28

**Authors:** Aziz Hamadah, Yoko Schreiber, Baldwin Toye, Sheryl McDiarmid, Lothar Huebsch, Christopher Bredeson, Jason Tay

**Affiliations:** 1 The Ottawa Hospital Blood and Marrow Programme, The Ottawa Hospital, Ottawa, Ontario, Canada; 2 Division of Infectious Diseases, The Ottawa Hospital, Ottawa, Ontario, Canada; Fred Hutchinson Cancer Center, United States of America

## Abstract

Empirical antibiotics at the onset of febrile neutropenia are one of several strategies for management of bacterial infections in patients undergoing Hematopoietic Stem Cell Transplant (HSCT) (empiric strategy). Our HSCT program aims to perform HSCT in an outpatient setting, where an empiric antibiotic strategy was employed. HSCT recipients began receiving intravenous antibiotics at the onset of neutropenia in the absence of fever as part of our institutional policy from 01 Jan 2009; intravenous Prophylactic strategy. A prospective study was conducted to compare two consecutive cohorts [Year 2008 (Empiric strategy) vs. Year 2009 (Prophylactic strategy)] of patients receiving HSCT. There were 238 HSCTs performed between 01 Jan 2008 and 31 Dec 2009 with 127 and 111 in the earlier and later cohorts respectively. Infection-related mortality pre- engraftment was similar with a prophylactic compared to an empiric strategy (3.6% vs. 7.1%; p = 0.24), but reduced among recipients of autologous HSCT (0% vs. 6.8%; p = 0.03). Microbiologically documented, blood stream infections and clinically documented infections pre-engraftment were reduced in those receiving a prophylactic compared to an empiric strategy, (11.7% vs. 28.3%; p = 0.001), (9.9% vs. 24.4%; p = 0.003) and (18.2% vs. 33.9% p = 0.007) respectively. The prophylactic use of intravenous once-daily ceftriaxone in patients receiving outpatient based HSCT is safe and may be particularly effective in patients receiving autologous HSCT. Further studies are warranted to study the impact of this Prophylactic strategy in an outpatient based HSCT program.

## Introduction

Bacteria are the major causative organisms of infections in the engraftment phase following hematopoietic stem cell transplant (HSCT). The use of peripheral blood stem cells and growth factors (G-CSF) shortens this phase, potentially improving infectious outcomes [1]. Nevertheless, neutropenic fevers occur in 90% and 80% of patients following allogeneic and autologous transplants respectively and [2]–[5] bacterial infections remain a leading cause of morbidity and mortality in HSCT recipients [6]–[8].

There are two predominant bacterial infection risk reduction strategies in HSCTs: 1) Prophylactic strategy where antibiotics are prescribed prior to the onset of neutropenia and 2) Empiric strategy where antibiotics are prescribed only at the onset of fever. Although there is evidence that bacterial prophylaxis is associated with improved clinical outcomes [9], its widespread use remains debated, given concerns regarding increased drug toxicity and antibiotic resistance [10]–[13]. Further, an empiric strategy may also be suboptimal as life-threatening septicemia may precede the development of fevers. This possibility becomes more worrisome when HSCT is performed in the outpatient setting. More recently, a pre-emptive strategy has been proposed [14] to optimize the benefits antibiotic use while limiting its complications.

The Ottawa Hospital Blood and Marrow Transplant Programme aims to perform HSCTs primarily in an outpatient setting. Prior to 01 Jan 2009, routine bacterial antibiotic prophylaxis was not prescribed. Our Programme was concerned about the rate of infection related mortality and upon review of the 2009 Infection Prevention Guidelines [15], changed our policy. After 01 Jan 2009, we implemented an intravenous Prophylactic antibiotic strategy where intravenous antibiotics were administered at the onset of neutropenia (absolute neutrophil count <0.5×10^9^/L) even if HSCT recipients remain afebrile. Our centre considered intravenous ceftriaxone in our Prophylactic strategy while patients remained in an outpatient HSCT setting for the following reasons: 1) once daily administration, 2) favorable safety profile, and 3) our institutional rate of quinolone resistance among gram negative bacteria is relatively high (20% resistance for *Escherichia coli*). Daily intravenous ceftriaxone was prescribed in an outpatient setting while standard dose intravenous piperacillin/tazobactam was given for inpatient recipients of HSCT.

We hypothesize that our prophylactic antibiotic strategy will reduce infection related HSCT complications while limiting the risk of C. difficile and frequency of empirical anti-fungal therapy. Specifically, we were most interested in whether our prophylactic strategy improves outcomes during the neutropenic phase of HSCT, where the recipient is most prone to bacterial infections (infection related mortality prior to engraftment). We sought to examine the impact of this strategy on the outcomes of our HSCT patients.

## Methods

### Patients

The Ottawa Hospital Blood and Marrow Programme maintain a database that prospectively collects transplant demographic and outcome data. Patients were eligible for our study if they received a HSCT at our Programme between 01 January 2008 and 31 December 2009. Patients receiving HSCT prior to 01 January 2009 received an empiric antibiotic strategy while patients receiving HSCT on and after 01 January 2009 received a prophylactic antibiotic strategy. All patients were included with stratification for allogeneic or autologous HSCT.

### Ethics

This data analysis was approved by our local Ethics Research Board. Personal health information (PHI) was gathered from the Ottawa Hospital Blood and Marrow Transplant Database. Participants of this database prospectively gave consent for their PHI to be stored and aggregated.

### Antibiotic Strategy

All patients received prophylaxis with acyclovir 400 mg twice daily and fluconazole 400 mg daily starting on the day of conditioning chemo/radiotherapy for viral and fungal prophylaxis. Further, trimethoprim/sulfamethazole prophylaxis was started post engraftment for all patients. With an empiric strategy, antibiotics were initiated upon diagnosis of febrile neutropenia. The initial choice of antibiotic was once daily 1 gram intravenous ceftriaxone for outpatients, while intravenous, standard dose with renal adjustments of piperacillin/tazobactam was prescribed for inpatients. Vancomycin was prescribed if a gram positive etiology was suspected. Subsequent antibiotic adjustment(s) was based on microbiologic and clinical setting. The antibiotic therapy(s) would continue until defervescence, the ANC is above 0.5×10^9^/L for 2 consecutive days, and in the absence of a microbiologically or clinically documented infection. Ceftriaxone or piperacillin/tazobactam were prescribed with the Prophylactic strategy but were initiated upon onset of neutropenia even in the absence of fever.

### Outcomes

Our primary outcome was infection related mortality prior to engraftment, defined as death in concurrence with a microbiologically or clinically documented infection within the pre-engraftment period. Our primary outcome of infection related mortality prior to engraftment was presented as the proportion of patients with the outcome of interest/total number of patients. Secondary outcomes include: all-cause mortality, infection related intensive care unit (ICU) admissions prior to engraftment, microbiologically documented infections prior to engraftment (MDI), clinically documented infections prior to engraftment (CDI), length of stay in hospital, need for escalation of antibiotics including the use of antifungals, incidence of Clostridium Difficile within 100 days of HSCT, Bearman Toxicity Scores [16] and resistance patterns of MDI (Table 1). Fungal infections were defined using standardized criteria [17] .Clinical outcomes were assessed for entry into our Programme database by a dedicated transplant advanced practical nurse. All patients were followed up for at least 100 day post HSCT or until death.

**Table 1:** Definition of Outcomes.

Outcomes	Definitions
**Neutropenia**	Absolute neutrophil count (ANC) <0.5×109/L or Leukocyte Count <1.0×109/L whichever occurs first.
**Engraftment**	ANC >0.5 109/L for 2 consecutive days.
**Microbiologically documented infections (MDI)**	The presence of1. Bloodstream infections, caused predominantly by bacteria and occasionally by fungi, without an identifiable non-hematogenous focus of infection, or2. Microbiologically proven site of infection (e.g. pneumonia, cellulitis, catheter related infection, urinary tract infection), with or without concomitant blood stream infection.We included any patient with a positive culture from blood, urine or pulmonary secretions (Endotracheal tube or bronchial washings, or sputum). Cultures would be included if they were positive for bacteria, fungi or virus (asymptomatic CMV viremias were not included). Coagulase negative staphylococci found in a single blood culture were excluded. Only Proven fungal infections were included, probable and possible were not. Virus detected by PCR or DFA from sterile site were included.
**Clinically documented infections (CDI)**	The presence of a site suggestive of infection even though the etiology of the infection has not been documented microbiologically.We included patients with clinically proven sites of infections (e.g. cellulitis, sepsis, imaging studies suggestive of pneumonia or neutropenic colitis). Fevers of Unknown origin were excluded.
**Infection Related Mortality**	Death associated with a concurrent MDI or CDI.
**Infection Related ICU Admission**	Admission to ICU associated with a concurrent MDI and/or CDI
**Length of Stay in Hospital**	Number of days from transplant (stem cell infusion) to discharge from hospital.
**Need for escalation of antibiotics**	Defined as any of the following changes:1. Switching from ceftriaxone to piperacillin/tazobactam2. Switching from piperacillin/tazobactam to Meropenem,3. Addition of Vancomycin4. Addition of antifungal therapy such as Caspofungin, Amphoterecin, Voriconazole or Posaconazole within the 1st 100 days of HSCT.
**Resistance patterns of MDI**	In vitro resistance or intermediate resistance to either piperacillin/tazobactum or 3rd generation cephalosporin.We also included any organism where meropenem or a non-beta-lactam agent would be the preferred treatment (e.g. Enterobacter, Citrobacter, etc.) within 100 days of HSCT.
**Bearman Toxicity Scores**	Maximum regimen related toxicity within the 1st 28 days of HSCT (ref see below)
**Monetary Cost**	The Ottawa Hospital anti-infective cost list was used. To calculate the cost we considered the price of antimicrobial drug given during hospitalization only. The cost of prophylactic trimethoprim/sulfamethoxazole, fluconazole and acyclovir was excluded.

PCR: Polymerase Chain Reaction; DFA: Direct Flourescence Antibody.

### Statistical Analysis

Statistical analysis facilitated by SAS version 9.1. Baseline characteristics between the 2 groups were compared with 2 sample tests, where categorical variables and continuous variables were compared using Chi-squared and Wilcoxan rank-sum tests respectively.

Given the limited censoring, we compared our primary outcome with Chi-squared test. However, to further appreciate patterns in mortality, all cause mortality was subjected survival analysis using Kaplan Meier method and potential differences between the 2 groups interrogated with log-rank tests. A multivariable stepwise logistic regression was applied to evaluate our primary outcome given the limited time-line. Potential confounding was controlled for by stratification by HSCT, namely autologous and allogeneic HSCT. Further, all baseline characteristics were considered as potential confounders and subjected to stepwise regression analyses for our primary outcome. All other secondary outcomes were summarized with proportions and compared with Chi-squared tests.

## Results

There were 238 HSCT performed at the Ottawa Hospital Blood and Marrow Programme between Jan 2008 to Dec 2009. One hundred and twenty seven HSCT patients received an empiric antibiotic strategy while 111 HSCT patients received a prophylactic strategy. Baseline characteristics and indications for HSCT between the groups were similar (Table 2). Median follow-up for the empiric and prophylactic groups were 461 and 213 days respectively. All but 2 deaths prior to engraftment in the prophylactic strategy group were attributable to infections, and no patients were lost to follow-up. The mean duration of neutropenia (time to engraftment) was 15.7 days and 14.8 days for the empiric and prophylactic groups respectively (p = 0.17).

**Table 2:** Baseline Characteristics.

	Empiric Strategy(n = 127)	Prophylactic Strategy(n = 111)	p value
**Mean age (years; range)**	47.9 (14.6–67.7)	50.1 (20.3–69.6)	0.22
**Gender**			
**Male**	77 (60.6%)	70 (63.1%)	0.64
**Female**	50 (39.4%)	41 (36.9%)	
**Transplant**			
**Allogeneic**	53 (41.7%)	41 (36.9%)	0.45
**Autologous**	74 (58.3%)	70 (63.1%)	
**Stem cell source**			
**Peripheral Blood**	114 (89.9%)	97 (87.4%)	
**Bone Marrow**	13 (10.2%)	12 (10.8%)	0.31
**Both**	0 (0%)	2 (1.8%)	
**Conditioning**			
**Myeloablative**	108 (85%)	102 (91.9%)	0.1
**Non-myeloablative**	19 (15%)	9 (8.1%)	
**Use of TBI**	24 (18.9%)	21 (18.9%)	1
**Conditioning regimen**			
**BEAM**	34 (26.8%)	32 (28.8%)	
**MEL200**	25 (19.7%)	20 (18%)	
**CY/TBI**	17 (13.4%)	16 (14.4%)	
**BU/CY**	16 (12.6%)	20 (18%)	0.82
**MEL/VP16/TBI**	7 (5.5%)	4 (3.6%)	
**BU/FLU**	24 (18.9%)	15 (13.5%)	
**Other**	4 (3.1%)	4 (3.6%)	
**HLA Matching**			
**Match Related Donor**	28 (52.8%)	22 (53.6%)	
**Matched Unrelated Donor**	25 (47.2%)	17 (41.4%)	0.37
**Haploidentical**	0 (0%)	2 (4.9%)	
**Diagnosis**			
**AML**	16 (12.6%)	18 (16.2%)	
**ALL**	3 (2.4%)	7 (6.3%)	
**MDS**	9 (7.1)	4 (3.6%)	
**MM**	25 (19.7%)	21 (18.9%)	
**NHL/HL**	57 (44.9%)	44 (39.6%)	
**CLL**	7 (5.5%)	2 (1.8%)	0.33
**CML**	1 (0.8%)	4 (3.6%)	
**AI**	5 (3.9%)	6 (5.4%)	
**AA**	0 (0%)	1 (0.9%)	
**Others**	4 (3.1%)	4 (3.6%)	

HLA: Human Leukocyte Antigen; AML: Acute myeloid leukemia; ALL: Acute lymphoid leukemia; MDS: Myelodysplasia; MM: Multiple myeloma; NHL: Non-Hodgkins lymphoma; CLL: Chronic lymphocytic Leukemia; CML: Chronic Myeloid Leukemia; AI: Autoimmune Diseases; AA: Aplastic Anemia; BEAM: BCNU, Etoposide, Ara-C, Melphalan; MEL200: Mephalan 200 mg/m^2^; CY/TBI: Cyclophosphamide/Total Body Irradiation; MEL/VP16/TBI: Melphalan/Etoposide/Total Boday Irradiation; BU/FLU: Busulfan/Fludarabine; TBI: Total Body Irradiation.

Infection related mortality prior to engraftment was similar between the two groups [3.6% (prophylactic) vs. 7.1% (empiric) p = 0.24], with 2 and 3 patients in the empiric and prophylactic group respectively who had >1 infective event. However, patients receiving autologous HSCTs had significantly less infection related mortality prior to engraftment in the prophylactic group as compared to the empiric group (0% vs. 6.8% p = 0.03) (Table 3). Further, our multivariable logistic regression model did not identify any significant association with any baseline characteristics or antibiotic strategy with our primary outcome. When limiting this analysis to recipients of autologous HSCT, there appears to be a trend towards improved infection related mortality prior to engraftment in the prophylactic group (p = 0.07).

**Table 3:** Clinical Outcomes.

Outcomes	All Patients			Patients undergoing autologous HSCT			Patients undergoing allogeneic HSCT		
	Empiric (n = 127)	Prophylactic (n = 111)	p value	Empiric (n = 74)	Prophylactic (n = 70)	p value	Empiric (n = 53)	Prophylactic (n = 41)	p value
**Infection Related Mortality pre-engraftment**	9 (7.1%)	4 (3.6%)	0.24	5 (6.8%)	0 (0%)	0.03	4 (7.5%)	4 (9.8%)	0.7
**Infection Related ICU admission pre-engraftment**	18 (14.2%)	9 (8.1%)	0.14	9 (12.2%)	2 (2.9%)	0.04	7 (13.2%)	6 (14.6%)	0.84
**All cause mortality at 100 days**	16 (12.6%)	10 (9.0%)	0.41	8 (10.8%)	3 (4.2%)	0.21	8 (15.0%)	7 (17.0%)	1
**All cause mortality pre-engraftment**	9 (7.1%)	6 (5.4%)	0.79	5 (6.8%)	0 (0%)	0.06	4 (7.5%)	6 (14.6%)	0.32
**MDI**	39 (30.7%)	16 (14.4%)	0.0029	20 (27%)	7 (10.0%)	0.0089	19 (35.8%)	9 (21.9%)	0.18
**BSI**	31 (24.4%)	10 (9.0%)	0.0017	18 (24.4%)	6 (8.6%)	0.01	13 (24.5%)	5 (12.2%)	0.19
**CDI**	42 (33.9%)	20 (18.2%)	0.0083	19 (26.4%)	9 (13%)	0.05	18 (34.0%)	7 (17.1%)	0.07
**Number of days alive and out of hospital to 100 days (sd)**	72.88(26.82)	80.01(21.42)	0.03	85.63(16.90)	77.70(25.94)	0.03	70.44(24.90)	66.15(26.83	0.43
**Fungal infections pre-engraftment**	0 (0%)	1 (0.9%)	0.47						
**Fungal infections at 100 days**	5 (3.9%)	3 (2.7%)	0.73						
**Viral infections prior to engraftment**	5 (3.9%)	3 (2.7%)	0.73						
**Viral infections at 100 days**	14 (11.0%)	9 (8.1%)	0.51						
**Mean Length of Stay (days)(sd)**	35.1±27.8	30.4±17	0.12						
**Escalation of antimicrobials** *	74 (58.3%)	57 (51.4%)	0.28						
**Incidence of Clostridium Difficile** *	6 (4.7%)	5 (4.5%)	0.94						
**Proportion of Resistant Bacteria in patients with MDI at 100days**	15 (38.5%)	10 (62.5%)	0.10						
**Bearman toxicity score**									
**0**	42 (36.8%)	33 (33.0%)							
**1–2**	54 (47.4%)	52 (52.0%)	0.79						
**>3**	18 (15.8%)	15 (15.0%)							

MDI: Microbiologically determined Infections; CDI: Clinically determined Infections; BSI: Blood Stream Infections; HSCT: Hematopoietic Stem Cell Transplantation;

* denominator includes all patients.

**Figure 1:**
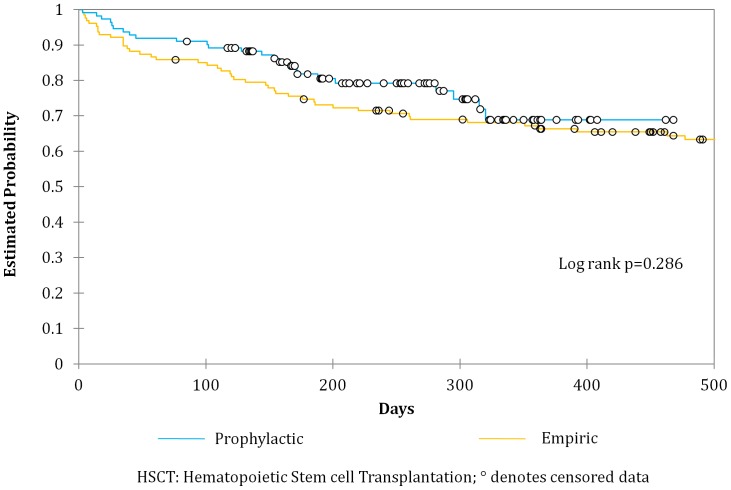
Overall survival of patients undergoing Hematopoietic Stem cell Transplantation receiving either a prophylactic or empiric antibiotic strategy.

**Figure 2:**
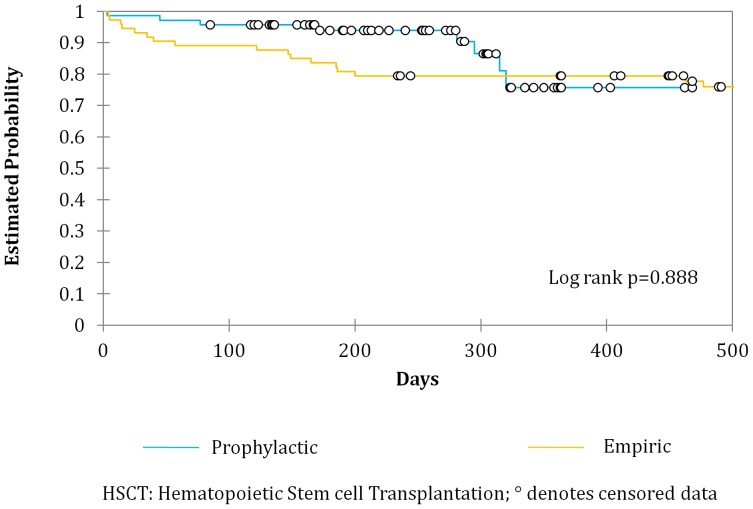
Overall Survival for patients undergoing allogeneic Hematopoietic Stem cell Transplantation receiving either a prophylactic or empiric antibiotic strategy.

**Figure 3:**
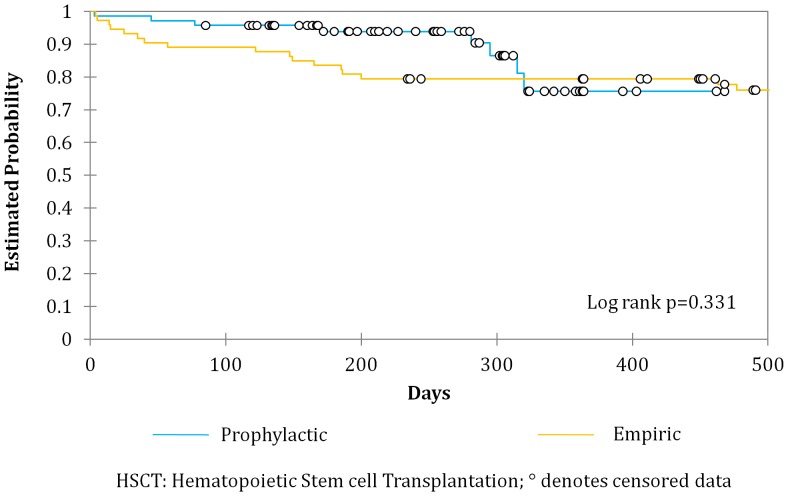
Overall Survival for patients undergoing autologous Hematopoietic Stem cell Transplantation receiving either a prophylactic or empiric antibiotic strategy.

Infection related ICU admissions prior to engraftment were similar between the two groups [8.1% (prophylactic) vs. 14.2% (empiric) p = 0.14) but there was a significant reduction of ICU admissions related to infection among the autologous transplants in the prophylactic group as compared to the empiric (12.2% vs. 2.9% p = 0.04). All cause mortality between both groups was similar (Figures 1–3).

The Bearman Toxicity scores (0, 1–2 or >2) were comparable in both cohorts (36.8% vs. 33%, 47.7% vs. 52% and 15.8% vs. 15%). Similarly, the length of stay in hospital (30.4 days vs. 35.1 days p = 0.12) was not different between the prophylactic and empiric cohorts (Table 3). However, there appears to be a trend in the mean length of hospital stay for patients who did not have an infection related death prior to engraftment where it was 30.3 days and 36.4 days in the prophylactic and empiric group respectively (p = 0.06).

Microbiologically (14.4% vs. 30.7% p<0.01) and clinically (18.2% vs. 33.9% p<0.01) documented infections prior to engraftment were significantly lower among the prophylactic group compared to the empiric. The majority of microbiologically documented infections were bacterial infections: 75% and 89% in the prophylactic and empiric group respectively. Similarly, blood stream infections in the pre-engraftment period were significantly lower in the prophylactic group compared to empiric group (9.0% vs. 24.4% p<0.01).

The need for escalation of antimicrobial therapy was not different between the two groups (51% vs. 58% p = 0.28). Further, the incidence of *Clostridium difficile* infection was not increased with the use of a prophylactic compared with an empiric strategy (4.5% vs. 4.7% p = 0.94) (Table 3).

There was no difference in the number of infections due to resistant bacteria with either a prophylactic or empiric strategy at 100 days post HSCT (34% vs. 35.7% p = NS). Further, the rate of proven fungal infections (2.7% vs. 4.7% p = 0.44) or viral infections at 100 days (14.4% vs. 15.7% p = 0.77) were similar over the two years (Table 3).

The mean (sd) number of days alive and out of hospital up to 100 days in the prophylactic and empiric groups were 80.01(21.42) days and 72.88(26.82) days respectively (p = 0.026). This effect was observed in patients receiving autologous HSCT [85.63(16.90) days and 77.70(25.94) days respectively] (p = 0.03) but not in patients receiving allogeneic HSCT [70.44(24.90) days and 66.15(26.83) days respectively] (p = 0.43).

The other clinical outcomes in patients receiving allogeneic HSCTs did not appear to have been attenuated by the implementation of a prophylactic strategy (Table 3).

## Discussion

Hematopoietic Stem cell Transplants (HSCTs) have traditionally been performed in an inpatient setting. However, with the introduction of PBSC mobilization, advent of better techniques, and supportive care, outpatient based HSCT have been adopted and shown to be feasible, safe and associated with cost reduction [18]–[21]. Although the incidence of infection is lower in the outpatient model compared to the in-patient, bacterial infections remain a major cause of morbidity and mortality in the first 100 days of HSCT, especially prior to neutrophil engraftment [22].

The use of prophylactic antibacterial strategy has been evaluated in the general oncology and HSCT setting, summarized by a large meta-analysis as well as more recent studies [9], [13], [23]–[25] suggest this strategy is beneficial in patients who are receiving cytotoxic therapy. Indeed, this has lead to the American Society for Blood and Marrow Transplantation to recommend quinolone antibacterial prophylaxis in patients undergoing HSCT [15]. However, these studies are either older or predominantly in a general oncology setting, where patients may have been at lower risk of infections when compared with recipients of HSCT. Further, when a HSCT setting was evaluated [9], the HSCT recipient was managed as an inpatient where prophylactic oral anti-bacterial agents were predominantly used. To our knowledge, there has been no direct literature that guides an outpatient based HSCT practice with a high institution rate quinolone resistance.

A systematic review of randomized controlled trials comparing quinolone prophylaxis with placebo or no intervention, or another antibiotic, for the prevention of bacterial infections in afebrile neutropenic patients showed that patients treated with quinolones have a non-significant increase in colonization by quinolone-resistant bacteria [14]. Further, there was no difference in the number of infections caused by pathogens resistant to quinolones. More recently, Liu el al. performed a retrospective review of their HSCT programme where either levofloxacin or non-levofloxacin containing antibiotic prophylaxis was prescribed to recipients of allogeneic HSCT. Their analysis suggests that 1) levofloxacin based prophylaxis results in more Gram-negative resistance organisms and 2) the presence of bacteremia is independently associated with greater length of hospital stay and 6 month mortality [26].

Several other strategies have been proposed to help predict early bacterial infection and/or sepsis, whereby antibiotics could be initiated earlier at the time of maximum risk. Firstly, Kimura el al. evaluated the utility of the Cumulative Area over the Neutropenia Curve index (c-D-Index), where the authors demonstrate that this calculated index may be an adjunct to help predict the risk of pulmonary infections, but not blood stream infections [27] in patients undergoing HSCT. Seeley et al. suggest that host response to infection is complex and is akin to a complex, non-linear system that can be analyzed using mathematical techniques of variability analysis [28]. In critical illness, there is a loss of “normal” variability of standard vital measures such as pulse and blood pressure. Indeed, the loss of heart rate variability has been shown to precede the development of the classic signs of infection [29].A pre-emptive antibacterial strategy in HSCT has been previously studied. Slavin et al. conducted a randomized trial of 153 patients undergoing either an autologous (52%) or allogeneic (48%) HSCT [30]. An empiric antibacterial strategy was compared with a pre-emptive strategy using intravenous cefepime in HSCT recipient with a primary outcome of reduction of incidence of fevers. Although their pre-emptive approach reduced the risk of bloodstream infections, this advantage did not translate into an appreciable clinical benefit as measured by days of hospitalization, time to engraftment, use of additional antimicrobial agents or mortality at 30 days.

The practice of HSCT has evolved over the last decade with an increasing number of patients transplanted [31]. This increase, in part could be the result of an increasing use of reduced-intensity HSCT, Further, the there has been an increased reliance on peripheral blood derived stem cells and cord blood. Advances in HLA typing techniques have afforded better transplant donor selection perhaps allowing more unrelated donor transplants [32]–[34]. Such changes in the host, donor, transplant graft sources and conditioning chemo-radiotherapy may alter the infectious risks for the HSCT recipient. Taken together, the optimal management of bacterial infections may not be well informed by older studies.

We performed prospective consecutive cohort study to evaluate our antibacterial strategy as previously described. Our intravenous prophylactic strategy was associated with decreased incidence of Microbiologic and Clinically Determined Infections, and associated with less infection related deaths and ICU admissions prior to engraftment in a patients undergoing autologous HSCT. Despite the use of prophylactic antibiotics, we did not document a difference in the incidence of resistant organisms, *C. difficile*, the need for escalation of antibiotics or drug cost between our 2 cohorts. Further the rate of fungal and viral infections were similar in both strategies both in the pre-engraftment period and at 100 days from HSCT.

The observed benefit of our intravenous prophylactic strategy in our autologous HSCT population remains unclear, with none observed in the allogeneic HSCT population. It can be argued that this study lacks sufficient power to detect a difference even in the higher risk allogeneic HSCT population. However, patients receiving autologous HSCT are more likely to remain in the outpatient setting as compared to patients receiving allogeneic HSCT due to the relative toxicities of the HSCT. An empirical antibiotic strategy in an outpatient setting relies heavily on patients' vigilance for signs and/or symptoms of infections and seeking timely review. Perhaps, this potential lack of vigilance can be partially negated by a prophylactic antibiotic strategy.

There are several limitations to our study. Firstly, an uncontrolled “before-after” study cannot account for secular trends that may have occurred over the time period (2 years) under review. We sought to minimize this bias by limiting the patient cohorts to just one year before and after the introduction of antibacterial strategy. It is possible that our HSCT infectious outcomes in 2008 are an outlier, artificially inflating our outcomes in 2009. Specifically the improved results in 2009 may be due to “regression to the mean” as opposed to the change in antibiotic policy. Further, we acknowledge that a 6.8% transplant mortality rate (predominantly due to infection related mortality) among recipients of autologous HSCTs in the pre-engraftment period could be considered higher than previously reported mortality rates [31]. However, we have previously published our outpatient HSCT experience between 1995 and 2006, demonstrating that the rates of infections in 2008 were comparable to the preceding years. This might suggest an “enhanced virulence” of the infective organisms or other transplant conditioning related toxicity in 2008. Nonetheless, the higher mortality rate in 2008 prompted a change in our Programme antibiotic strategy. As a result of this policy change, the mortality rates in the pre-engraftment period after changing the antibiotics strategy was not only much better than the previous year (2008) but even better than the reported mortality at our centre in the years between 1995 to 2006 [22]. Second, our study reflects a single center experience where HSCTs are performed predominantly in an outpatient setting. Indeed, local antibiotic resistance patterns and geographic setting have to be considered when adopting an anti-bacterial strategy. Third, the duration of the follow-up (1–2 years) might be inadequate to assess for the emergence of antimicrobial resistance. Nevertheless, it is encouraging that our intravenous prophylactic anti-bacterial strategy did not result in an increase in resistant organisms in its first year of implementation. Further close monitoring for the development of resistance is prudent with the use of this intravenous prophylactic strategy. Finally, our study assessed a heterogeneous group of patients where the risk of infection related complications may be different; where differing strategies for different risk categories may be preferable. Nonetheless, we demonstrate that our intravenous prophylactic strategy is safe in patients undergoing both autologous and allogeneic HSCTs. Further, autologous HSCT recipients may specifically benefit from an intravenous prophylactic anti-bacterial strategy, leading to a reduction of infection related mortality when compared to an empiric strategy.

We did not demonstrate a statistical benefit in allogeneic HSCT recipients, perhaps as a result of a lack of statistical power to detect a difference (n = 94). Ultimately, the choice between prophylactic intravenous antibiotic and prophylactic oral broad-spectrum antibiotics is complex and may include factors such as HSCT Programme structure, antibiotic resistance patterns, patient preference and cost. Unfortunately, the assessment of this choice is beyond the scope of this retrospective analysis.

In conclusion, an intravenous prophylactic anti-bacterial strategy in an outpatient based HSCT programme at onset of neutropenia is safe and feasible, with a potential to reduce infection related morbidity and mortality.
